# Acute peritonitis caused by a giant appendicolith: A rare case report and a literature review

**DOI:** 10.1016/j.ijscr.2025.111198

**Published:** 2025-03-26

**Authors:** Maryam Maghbool, Babak Samizadeh, Sepehr Ramezanipour

**Affiliations:** a Clinical Research Development Unit of Valiasr Hospital, Fasa University of Medical Sciences, Fasa, Iran; b Fasa University of Medical Sciences, Fasa, Iran

**Keywords:** Appendicolith, Peritonitis, Appendicitis, Appendectomy, Acute abdomen, Case report

## Abstract

**Introduction:**

Giant appendicoliths, which are calcified deposits larger than 2 cm found in the appendix, are uncommon and often linked to appendicitis, as well as complications like perforation or abscess formation. The occurrence of a giant appendicolith leading to peritonitis without accompanying appendicitis is rare, presenting a distinct diagnostic and therapeutic challenge.

**Presentation of case:**

A 77-year-old male with beta-thalassemia minor came in with acute pain in the right lower quadrant, along with nausea and vomiting. Upon examination, he showed signs of peritoneal irritation, including rebound tenderness and guarding. Laboratory tests indicated mild leukopenia and normal inflammatory markers. Imaging studies identified a 5 cm appendicolith and localized free fluid suggestive of perforation, along with signs of superimposed peritonitis. Surgical intervention revealed a distended appendix containing the giant appendicolith and an ileocecal perforation, but histopathological analysis showed no evidence of acute appendicitis. The patient underwent an appendectomy and repair of the perforation, resulting in an uneventful recovery.

**Discussion:**

Giant appendicoliths can lead to significant mechanical irritation and complications such as perforation, even in the absence of the typical inflammatory response associated with appendicitis. The diagnostic difficulty arises from the lack of fever and elevated inflammatory markers, which are usually present in cases of acute appendicitis.

**Conclusion:**

Giant appendicoliths should be included in the differential diagnosis for acute abdominal pain, even when appendicitis is not evident. This case highlights the necessity of thorough approaches for accurate diagnosis and effective treatment.

## Highlights

• Acute peritonitis caused by a giant appendicolith without evidence of acute appendicitis is a rare phenomenon.
• This phenomenon presents a diagnostic challenge.
• Comprehensive approaches, including imaging, surgical intervention, and histopathological analysis, was pivotal for accurate diagnosis and treatment.
• This case highlights the importance of considering appendicoliths in the differential diagnosis of acute abdominal pain, even in the absence of typical appendicitis signs

## 1 Introduction

Secondary peritonitis is a life-threatening surgical emergency requiring prompt source control, antibiotic therapy, and intensive care for optimal outcomes [1]. Approximately 7–10 % of all emergency room referrals are for acute abdominal pain, with acute appendicitis as one of the most common causes of lower abdomen pain [2]. Perforation occurs in 20–30 % of Acute appendicitis cases [3]. Although, appendicitis and appendicular perforation are common etiologies of peritonitis, the severity of the abdominal infection, the site of perforation, and the patient's overall condition affect clinical outcomes [4]. Rare cases involving atypical pathophysiological mechanisms, such as appendicolith, challenge clinical decision-making. Appendicolith, a calcified fecal deposit, can obstruct the appendix and lead to appendicitis, perforation, and recurrent complications [5,6]. About 40 % of acute appendicitis cases identified by CT show appendicolith, which is linked to a higher risk of complicated acute appendicitis and perforation [7]. However, appendicoliths may also be incidental findings in asymptomatic patients [5]. This case report presents a 77-year-old male with peritonitis due to a giant 5 cm appendicolith.

This case report presents a 77-year-old male with peritonitis due to a giant 5 cm appendicolith. To the best of our knowledge, appendicoliths larger than 2 cm are very rare, and this report describes the largest known appendicolith. This study highlights the need to recognize appendicolith as a potential cause of perforation and secondary peritonitis, even in the absence of appendicitis.

## 2 Presentation of case

**Fig. 1:**
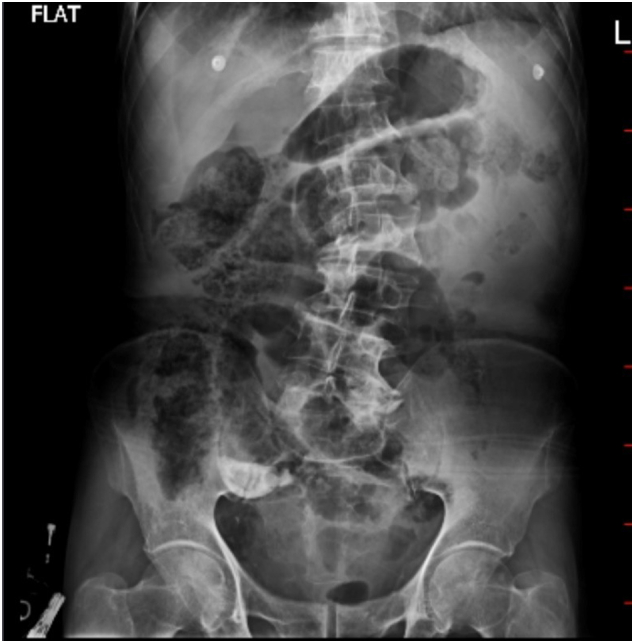
Abdominal X-ray; calcifications in the right lower quadrant.

**Fig. 2:**
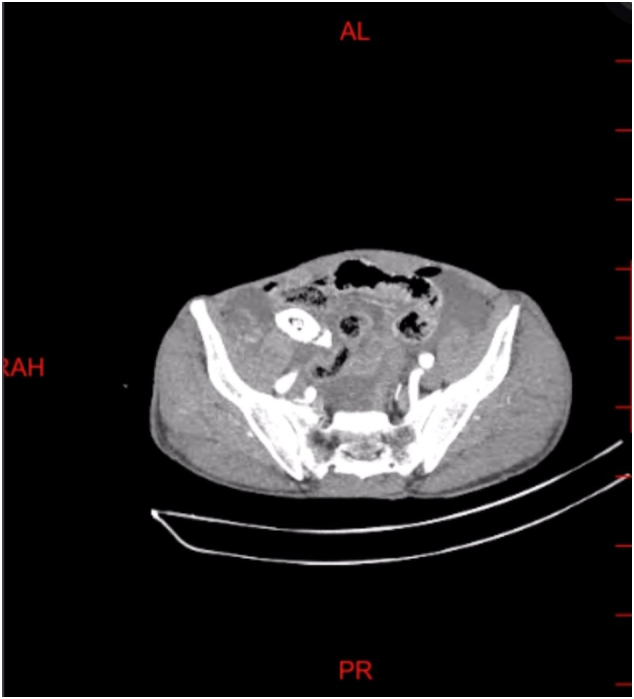
Axial view of abdominopelvic CT-scan with IV contrast.

**Fig. 3:**
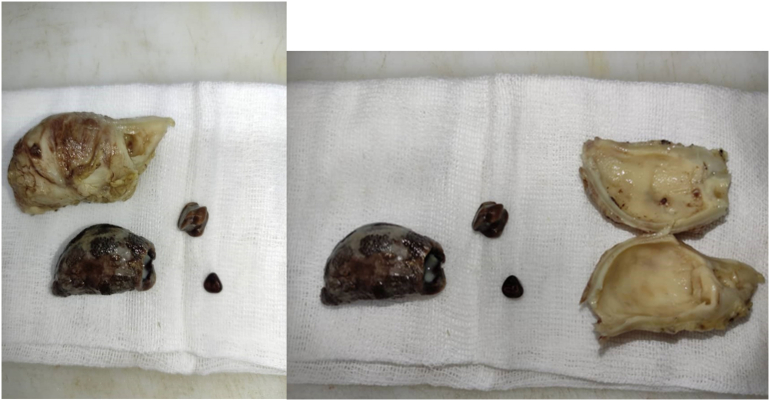
Gross specimen of the appendix showing multiple appendicoliths, including a large 5 cm appendicolith.

**Fig. 4:**
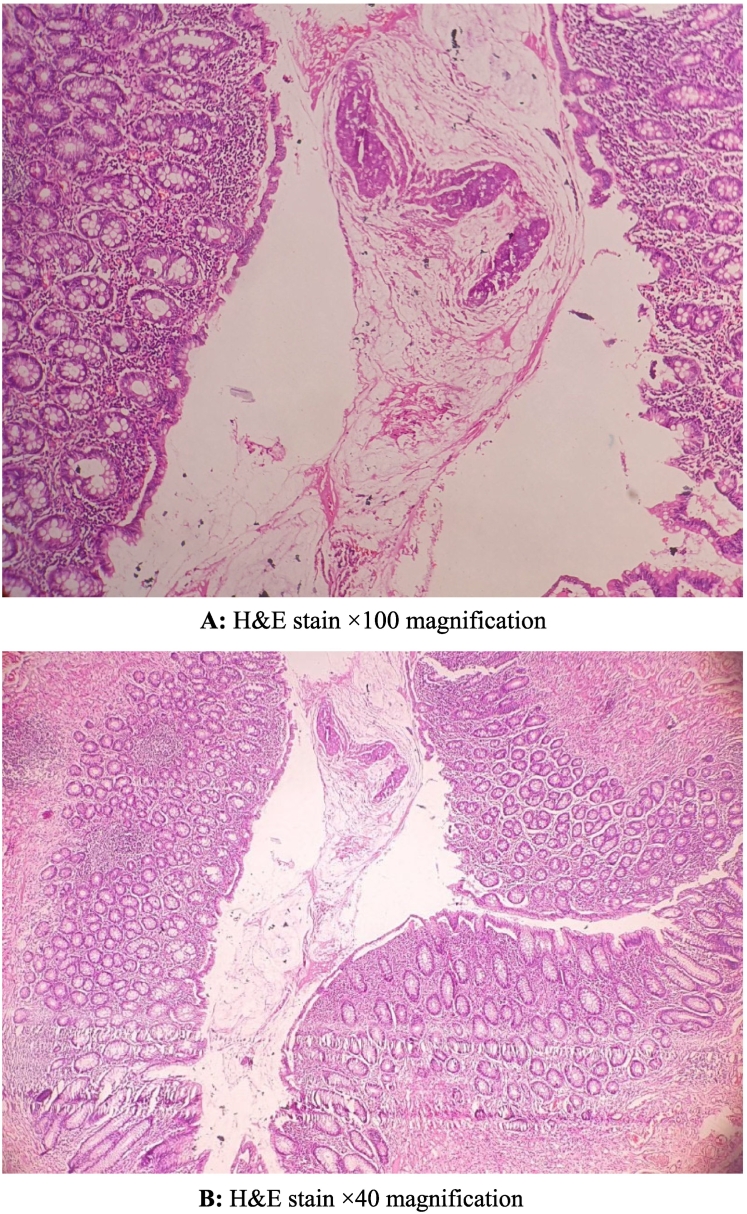
Histopathological examination confirmed the absence of acute appendicitis.

**Fig. 5:**
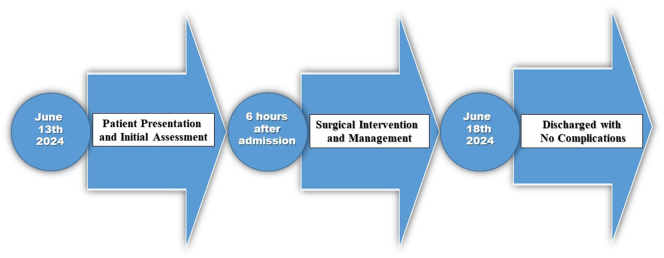
A summary of patient's clinical course.

In June 2024, a 77-year-old male with a history of beta-thalassemia minor presented to the emergency department with intense, widespread abdominal pain that started a few hours prior. The pain was severe, with no relief in any position, and unrelated to food. He also reported nausea, vomiting, constipation, and decreased appetite but denied fever, chills, or urinary symptoms. He had no previous surgeries or chronic conditions. On examinations, the patient was alert, with a heart rate of 98 beats per minute, blood pressure of 100/70 mmHg, respiratory rate of 23 breaths per minute, temperature of 37.6 °C, and oxygen saturation of 93 % on room air. Abdominal examination revealed generalized tenderness, guarding and rebound tenderness, suggesting peritoneal irritation. Bowel sounds were diminished, and no palpable masses or organomegaly were found. Initial laboratory tests revealed mild leukopenia (WBC 2.9 × 10^3^/μL), a hemoglobin level of 10.8 g/dl, and normal kidney and liver function tests. There were no visible signs of significant inflammation; the C-reactive protein level was 0.1 mg/dl, and the Erythrocyte sedimentation rate was 3 mm/h. Coagulation markers were within normal range. An abdominal X-ray showed prominent bowel loops with gaseous distension and calcifications in the right lower quadrant suggestive of appendicolith (Fig. 1). In the ultrasound, moderate free fluid was found in the pelvic cavity and bowel wall thickening. CT scan revealed a large oval calcified structure measuring 35 mm in length and 18 mm in AP diameter in the right lower quadrant, suggestive of a large appendicolith. Mild free air and fluid were present in the abdominopelvic cavity, suggesting possible pneumoperitoneum due to perforation. Additionally, mild circumferential wall thickening of small bowel loops, along with diffuse mesenteric haziness and fat stranding, indicated superimposed peritonitis (Fig. 2). Given these findings, the patient underwent an emergency laparotomy by an expert general surgeon. During surgery, the surgical team found a severely distended appendix (6 × 3 cm) filled with several appendicoliths, the largest measuring 5 × 4 × 2 cm (Fig. 3). They also noted a small perforation in the ileocecum, likely caused by mechanical irritation. There was localized peritoneal contamination, but no signs of significant inflammation or abscess formation. The perforation was repaired using primary sutures (enterorrhaphy), and an appendectomy was performed. Histopathological examination confirmed the absence of acute appendicitis. The appendix had smooth mucosa without significant inflammatory infiltrates in the submucosa or muscularis layers. The lumen contained a large calcified appendicolith with concentric calcification indicative of chronic stasis. Localized fibrosis was noted, but no ulceration, necrosis, or transmural inflammation was present (Fig. 4). Postoperatively, the patient was placed on broad-spectrum antibiotics and monitored in the ICU. His condition improved significantly, and he was discharged on the fifth postoperative day with no complications, and his clinical status remained stable during the follow-up period to date (Fig. 5).

## 3 Methodology

We searched for “(Appendicolith [tiab] OR appendiceal calculus [tiab] OR appendiceal stone [tiab] OR fecalith [tiab] OR coprolith [tiab] OR appendicolithiasis [tiab] OR appendiceal concretion [tiab] OR intraluminal calculus [tiab] OR appendiceal lithiasis [tiab] OR appendiceal bezoar [tiab] OR vermiform appendix stone [tiab]) AND (Peritonitis [tiab] OR intra-abdominal infection [tiab] OR perforation [tiab] OR acute abdomen [tiab] OR abdominal emergency [tiab] OR acute abdominal pain [tiab] OR appendicitis [tiab] OR appendectomy [tiab] OR laparotomy [tiab]) AND (Case Report)” in the PubMed database. Relevant articles were retrieved in full text. The bibliographic search was completed on March 6, 2025. The work has been reported in line with the SCARE 2023 criteria [8].

## 4 Discussion

### 4.1 Appendicoliths pathophysiology

Appendicoliths are calcified masses of stool and mineral deposits that can obstruct the appendiceal lumen, increasing intraluminal pressure and leading to venous congestion, ischemia, and potential perforation [9]. Wangensteen and Dennis (1939) initially proposed that such obstructions trigger appendicitis through secretion pooling, pressure buildup, and bacterial proliferation [10]. However, incomplete obstruction may allow drainage, preventing rapid pressure accumulation and acute inflammation. While appendicoliths are commonly associated with appendicitis, not all cases lead to inflammation. Large appendicoliths may develop gradually, allowing luminal adaptation and reducing the likelihood of ischemia [11]. This phenomenon was observed in a 67-year-old transplant patient with a >2 cm appendicolith who remained asymptomatic with a normal appendix on CT [11].

Additionally, some giant appendicoliths lead to a walled-off process rather than diffuse peritonitis. One case described an appendiceal inflammatory mass resulting from a slow perforation by a 2.5 cm appendicolith, forming a retroperitoneal abscess without systemic inflammation [12]. Appendicoliths are also detected incidentally in up to 32 % of healthy individuals undergoing imaging [13]. A four-year study found no cases of appendicitis among patients with incidental appendicoliths, highlighting that many remain asymptomatic despite their presence [5]. Even large stones can remain silent. Previous research has noted that most appendicoliths are asymptomatic, though they carry a potential risk for complications [14]. While they carry a potential risk for complications, factors such as infectious agents, genetic predisposition, and environmental influences (climate, air pollution, smoking) also contribute to appendicitis development [[15], [16], [17], [18], [19]].

In our case, the giant appendicolith directly caused ileocecal perforation without preceding appendicitis. Histology confirmed the absence of inflammation, emphasizing that mechanical perforation can occur independently of an inflammatory response. This rare presentation may be explained by chronic appendicolith formation, gradual luminal adaptation, and potential bacterial sequestration within the stone's structure.

### 4.2 Appendicoliths characteristics

Appendicoliths are classified based on composition, structure, and size. Forbes and Lloyd-Davies (1966) categorized them into fecal pellets, partially calcified fecaliths, and true calculi [20]. More recent studies classify them by hardness, demonstrating that higher calcium content does not necessarily correlate with appendicitis severity. Notably, harder appendicoliths often exhibit layered structures [21], which may limit bacterial exposure and reduce acute inflammation, though their clinical significance remains uncertain. The most practical classification is by size. The term “giant appendicolith” applies to stones >2 cm in diameter, though most appendicoliths are much smaller (typically a few millimeters to 1 cm) [22]. Giant appendicoliths are rare, with fewer than 20 cases reported in the literature [23]. Our case, featuring a 5 cm appendicolith, represents the largest known example, surpassing prior reports of 3.5–4.5 cm stones [11,24]. While larger appendicoliths have a higher likelihood of causing obstruction and perforation, they do not always lead to appendicitis [25]. Several studies highlight the relationship between appendicolith size, inflammatory markers, and perforation risk. Akira Kubota et al. demonstrated that among appendicitis patients, those with appendicoliths >10 mm and CRP >10 mg/dL had a higher risk of perforation, often requiring surgical intervention [26]. However, complicated appendicitis can still occur in patients with normal WBC and CRP levels, reinforcing the importance of imaging-based assessment [27].

Appendicoliths vary in number and location. They can be single or multiple, with multiple stones and proximal positioning at the appendiceal base being associated with a higher risk of appendicitis [13]. Although the exact reason why some appendicoliths lead to appendicitis while others remain asymptomatic is still unknown, factors such as size, location, and the presence of multiple stones may contribute to the risk. Clinically, appendicoliths are implicitly classified as either incidental or associated with appendicitis. Incidental appendicoliths are detected on imaging in patients without appendicitis symptoms, often during evaluation for unrelated conditions. Notably, most incidental appendicoliths do not progress to appendicitis in short-term follow-up [5]. A distinct classification is the “dropped appendicolith”, referring to a stone that escapes the appendix due to perforation or surgery, potentially leading to abscess formation [28]. While extraluminal appendicoliths are commonly linked to perforation and abscess formation [7], our case differs as the appendicolith remained within the appendix, contributing to ileocecal perforation via chronic pressure rather than acute inflammation. This highlights the importance of assessing both size and location, as giant appendicoliths may cause complications even in the absence of active inflammation.

### 4.3 Diagnostic challenges

Imaging, particularly computed tomography (CT), plays a crucial role in diagnosing appendicoliths and their complications [5,11]. In our case, abdominal CT identified a 5 cm appendicolith, an ileocecal defect, and free fluid suggestive of localized peritonitis, despite normal inflammatory markers. This highlights the importance of radiological evaluation in atypical cases, where standard laboratory markers may not reflect disease severity. Appendicoliths can be detected using CT, ultrasonography (USG), and abdominal X-rays. Some cases present as “appendiceal colic,” with recurrent right lower quadrant pain but no true appendicitis [29]. Additionally, appendicoliths may mimic urolithiasis, as both conditions can present with abdominal pain, leukocytosis, and even hematuria, making differentiation challenging [14].

X-rays may reveal a radiopaque structure in the right lower quadrant, sometimes mistaken for renal or gallstones [30]. USG detects appendicoliths as echogenic foci with shadowing, but large stones may be obscured by bowel gas. One case demonstrated how USG identified a 16 mm appendicolith, while CT revealed a larger 18 mm stone within a distended appendix [31]. CT remains the most effective imaging modality, as it can distinguish appendicoliths from ureteral stones, preventing misdiagnosis and ensuring appropriate management [14]. In this case, the patient presented with acute abdominal pain, nausea, and signs of peritonitis but lacked fever, leukocytosis, and elevated inflammatory markers creating diagnostic uncertainty. CT showed a giant calcified appendicolith, and moderate free fluid suggestive of localized peritonitis.

CT is particularly effective in detecting appendicoliths even in the absence of inflammation, improving diagnostic accuracy and surgical decision-making [6]. Studies indicate that appendicoliths appear in 38.7 % of confirmed appendicitis cases but only 4.4 % of non-appendicitis patients [7]. Although some suggest appendicoliths are 100 % specific for appendicitis, other research demonstrates their presence without inflammation. CT has a sensitivity of 65 %, a specificity of 86 %, and a 74 % positive predictive value, indicating that an appendicolith alone does not confirm active appendicitis [32]. This complexity was evident in our case, where CT suggested perforation, yet histopathology revealed an uninflamed appendix, emphasizing the distinction between mechanical and inflammatory complications.

Histopathology remains invaluable in post-operative assessment. While some studies report mucosal ulceration and neutrophilic infiltration due to an appendicolith [31], our patient's histology showed no neutrophilic infiltrate, confirming that the perforation was mechanical rather than inflammatory. Hennenberg et al. reported a 4.5 cm appendicolith with severe inflammation, minimal perforation, and adhesions. Histopathology confirmed ulcero-phlegmonous appendicolithiasis and periappendicitis, demonstrating its role in inflammation [24]. In contrast, our larger 5 cm appendicolith caused no significant inflammation, highlighting the variability in appendicolith-related complications.

Ultimately, correlating imaging, clinical presentation, and histopathology is essential, especially in atypical cases. In our patient, the CT finding of a giant appendicolith with localized free fluid indicated a surgical emergency, even though classical signs of appendicitis were absent. This case highlights the need for careful interpretation of imaging findings in patients with giant appendicoliths to ensure timely and appropriate intervention.

### 4.4 Management strategies in appendicolith-related complications

Secondary peritonitis is an acute infection of the peritoneum resulting from a breach in the gastrointestinal or other visceral organs. Often due to perforations, from conditions like diverticulitis, appendicitis, or cholecystitis, as well as traumatic or iatrogenic injuries. Management typically involves surgery to remove the infectious source, along with intensive care, fluid resuscitation, antimicrobial therapy, and nutritional support [33]. In this case, laparotomy was performed due to physical examination and imaging findings suggestive of peritonitis. The decision for appendectomy and primary repair of the perforation, rather than bowel resection, was made due to the absence of severe inflammation and the localized pathology, allowing for a targeted approach. However, the literature highlights a debate over surgical intervention vs. nonoperative management (NOM) for the treatment of appendicoliths.

A study found a higher NOM failure rate in appendicitis patients with appendicoliths (37 %) compared to those without (10 %, *P* < .05). While appendicoliths increase the risk of NOM failure, they are not considered an absolute contraindication, emphasizing individualized treatment [34]. A recent meta-analysis of 12 studies (814 pediatric patients) found that appendicoliths do not affect initial success rates of NOM in complicated appendicitis but significantly increase recurrence risk. In simple appendicitis, NOM had lower success rates with appendicoliths, supporting early appendectomy. Due to the high recurrence risk, interval appendectomy is recommended after NOM [35]. Although surgical intervention is the regular approach, there have been reported appendicoliths extracted using the endoscopic method [36]. Moreover, there are reports of appendicoliths being managed conservatively without surgery, primarily when discovered incidentally in asymptomatic patients or those with significant operative risks [11].

## 5 Conclusion

In summary, we presented a rare case of a giant appendicolith measuring 5 cm, causing localized ileocecal perforation and secondary peritonitis without associated appendicitis. Histopathological analysis confirmed the absence of inflammation, underscoring the unique pathophysiological mechanism of chronic mechanical irritation. This case underscores the importance of integrating clinical, radiological, surgical, and pathological findings to ensure accurate diagnosis and optimal management, encouraging clinicians to recognize isolated appendicolith as a potential cause of complications.

## Author contribution

Sepehr Ramezanipour [Writing - Original Draft, Review, and editing and submission].

Maryam Maghbool [Conceptualization, Methodology, Data Curation, Project Administration].

Babak Samizadeh [Conceptualization, Methodology, Data Curation, and Project Administration].

## Consent

Written informed consent was obtained from the patient, for publication of this case report and accompanying images. A copy of the written consent is available for review by the Editor-in-Chief of this journal on request.

## Ethical approval

This study was conducted in full compliance with ethical standards and received approval from the Ethics Committee of Fasa University of Medical Sciences under the ethical code IR.FUMS.REC.1403.121.

## Guarantor

Maryam Maghbool.

## Research registration number

Not applicable.

## Declaration of Generative AI and AI-assisted technologies in the writing process

During the preparation of this work, the authors only used artificially intelligent proofing tools to enhance the lingual experience for the readers. No other generative AI and AI-assisted technology was used in the writing process. After using the tool, the authors reviewed and edited the content as needed and take full responsibility for the content of the publication.

## Funding

The authors declare that no financial support was received for the research, authorship, or publication of this article.

## Conflict of interest statement

None.
